# Transmission of B.1.617.2 Delta variant between vaccinated healthcare workers

**DOI:** 10.1038/s41598-022-14411-7

**Published:** 2022-06-21

**Authors:** Steven A. Kemp, Mark T. K. Cheng, William L. Hamilton, Kimia Kamelian, The Indian SARS-CoV-2 Genomics Consortium (INSACOG), Himanshu Chauhan, Tanzin Dikid, Hema Gogia, Hemlata Lall, Kalaiarasan Ponnusamy, Kaptan Verma, Mahesh Shanker Dhar, Manoj K. Singh, Meena Datta, Namita Soni, Namonarayan Meena, Preeti Madan, Priyanka Singh, Ramesh Sharma, Rajeev Sharma, Sandhya Kabra, Sattender Kumar, Swati Kumari, Uma Sharma, Urmila Chaudhary, Sridhar Sivasubbu, Vinod Scaria, Chand Wattal, J. K. Oberoi, Reena Raveendran, S. Datta, Saumitra Das, Arindam Maitra, Sreedhar Chinnaswamy, Nidhan Kumar Biswas, Ajay Parida, Sunil K. Raghav, Punit Prasad, Apurva Sarin, Satyajit Mayor, Uma Ramakrishnan, Dasaradhi Palakodeti, Aswin Sai Narain Seshasayee, K. Thangaraj, Murali Dharan Bashyam, Ashwin Dalal, Manoj Bhat, Yogesh Shouche, Ajay Pillai, Priya Abraham, Varsha Atul Potdar, Sarah S. Cherian, Anita Sudhir Desai, Chitra Pattabiraman, M. V. Manjunatha, Reeta S. Mani, Gautam Arunachal Udupi, Vinay Nandicoori, Karthik Bharadwaj Tallapaka, Divya Tej Sowpati, Sujit Singh, Partha Rakshit, Anurag Agrawal, Christopher J. R. Illingworth, Ravindra K. Gupta

**Affiliations:** 1 Cambridge Institute of Therapeutic Immunology & Infectious Disease (CITIID), Cambridge, UK; 2 Department of Medicine, University of Cambridge, Cambridge, UK; 3 National Microbiology Laboratory, Public Health Agency of Canada, Winnipeg, MB Canada; 4 National Centre for Disease Control, Delhi, India; 5 CSIR Institute of Genomics and Integrative Biology, Delhi, India; 6 Garscube Campus, MRC - University of Glasgow Centre for Virus Research, 464 Bearsden Road, Glasgow, UK; 7 MRC Biostatistics Unit, University of Cambridge, East Forvie Building, Forvie Site, Robinson Way, Cambridge, UK; 8 Department of Applied Mathematics and Theoretical Physics, University of Cambridge, Cambridge, UK; 9 Africa Health Research Institute, Durban, South Africa; 10 Jeffrey Cheah Biomedical Centre, Cambridge, CB5 8UB UK; 11 Department of Biotechnology, Delhi, India

**Keywords:** Medical research, Epidemiology, Infectious diseases

## Abstract

Breakthrough infections with SARS-CoV-2 Delta variant have been reported in doubly-vaccinated recipients and as re-infections. Studies of viral spread within hospital settings have highlighted the potential for transmission between doubly-vaccinated patients and health care workers and have highlighted the benefits of high-grade respiratory protection for health care workers. However the extent to which vaccination is preventative of viral spread in health care settings is less well studied. Here, we analysed data from 118 vaccinated health care workers (HCW) across two hospitals in India, constructing two probable transmission networks involving six HCWs in Hospital A and eight HCWs in Hospital B from epidemiological and virus genome sequence data, using a suite of computational approaches. A maximum likelihood reconstruction of transmission involving known cases of infection suggests a high probability that doubly vaccinated HCWs transmitted SARS-CoV-2 between each other and highlights potential cases of virus transmission between individuals who had received two doses of vaccine. Our findings show firstly that vaccination may reduce rates of transmission, supporting the need for ongoing infection control measures even in highly vaccinated populations, and secondly we have described a novel approach to identifying transmissions that is scalable and rapid, without the need for an infection control infrastructure.

## Introduction

As of March 2022, SARS-CoV-2 has affected more than 459 million people and caused more than 6 million deaths worldwide^1^. The ongoing emergence of viral mutations has led to concerns regarding vaccine efficacy (VE) and prospects of transmission amongst fully-vaccinated patients. For much of 2021 the Delta (B.1.617.2) variant of concern (VOC) was the predominant strain of SARS-CoV-2 worldwide, replaced at the start of 2022 by Omicron.

Numerous VE studies have reported that both the BNT162b2 and ChAdOx1 nCoV-19 vaccines have lower efficacy against the Delta variant, compared to Wuhan-1 or Alpha variants. Initial case–control studies of VE in India indicated that for mild symptomatic infection with Delta, after two doses of either mRNA or adenovirus-based vaccine platforms, VE was approximately 50%^2^. However, VE for moderately-severe and severe disease is estimated as 88.0%–93.4%^3,4^ when vaccinated with the BNT162b2 vaccine. For any asymptomatic or asymptomatic infection, VE has been reported to vary between 51.9% when infected ≤ 14 days after 2nd-dose, to 93.7%^4–6^ when infected > 15 days after a 2nd-dose of the ChAdOx1 nCoV-19 vaccine.

Breakthrough infections of the Delta variant have been reported in vaccine recipients, as well as re-infection of individuals infected with previously circulating variants^7–10^. In India, vaccination of health care workers (HCWs) started in early 2021 with the ChAdOx-1 vaccine. We previously reported 155 occurrences of vaccine breakthrough infections^5^ amongst those HCW, all of whom had one or two doses of the vaccine, with the majority vaccinated > 14 days prior to symptomatic presentation.

Existing literature on viral loads is conflictive, Some suggest that there are similar viral loads between vaccinated and unvaccinated^11,12^ patients, but there is a steeper decay^13,14^ over time in vaccinated persons. Conversely, there are also reports of lower viral loads in breakthrough infections with Alpha^15^ and Delta^16^ variants. However, it is clear from contact tracing data that there is likely a reduced probability of household transmission from individuals who have been vaccinated^17^.

A variety of studies have highlighted the need to better understand and prevent SARS-CoV-2 transmission in hospital settings^18^. Newly infected patients pose a risk both to other patients and to health care workers^19^, and infection of patients by HCWs is potentially a rarer event, though HCWs do pose a risk to each other^20^. Distinct approaches have been taken to reduce the risk to HCWs of infection at work; a study of the use of FFP3 respirators by HCWs in one hospital showed that these provided at least a 52% improvement in protection against infection compared to fluid resistant surgical masks^21^. Similarly, HEPA filters have suggested as a further measure in protecting patients and staff^22^ and use of antiviral surface coatings has also been considered^23^. The use of viral genome sequencing has prompted the development of new approaches for the rapid assessment of cases of hospital infection^24,25^. Genome sequencing has demonstrated benefit in health care settings^26–28^ by determining circulating strains and affording insights into ongoing transmission events.

Here we evaluated cases of SARS-CoV-2 infection among vaccinated and unvaccinated HCW in two major Indian hospitals. Applying in silico approaches to genomic and epidemiological data we identified potential transmission networks involving HCWs. Our results suggest that transmission of the Delta variant of SARS-CoV-2 may have occurred from and between doubly-vaccinated HCWs.

## Results

### ChAdOx1 nCoV-19 breakthrough infection

All symptomatic HCWs in two hospitals who had received one or two doses of the ChAdOx1 nCoV-19 adenoviral vaccine (AZD1222), in addition to a set of unvaccinated HCWs, underwent RT-PCR testing within two days of commonly identified SARS-CoV-2 symptoms, including fever, a continuous cough and anosmia (Table 1) onset as part of a hospital staff symptomatic testing program. From these cases we identified breakthrough infection in staff who had received two doses of the vaccine. In hospital A, there were 81 breakthrough infections amongst 1100 HCWs, and in hospital B, 32 infections amongst 4000 HCWs, as previously reported^5^.

**Table 1:** Symptom prevalence amongst vaccinated HCWs in both hospitals.

Symptoms	Number of cases	% of total
Asymptomatic	2	1.8
Weakness	2	1.8
Nausea and/or vomiting	3	2.7
Dyspnoea	3	2.7
Congestion	5	4.4
Diarrhoea	5	4.4
Headache	12	10.6
Anosmia and/or ageusia	16	14.2
Sore throat	32	28.3
Myalgia (including backache)	23	20.4
Cough	49	43.4
Fever	93	82.3

Among the 113 cases from hospitals A and B, 12.4% were administrative staff, 31.9% were nurses, 40.7% were primary physicians, 7.1% were paramedics and 3.5% were pharmacists. The remaining 3.5% of breakthrough infections consisted of medical interns (1.8%), and laboratory workers (1.8%). The median interval between receiving a second vaccination and date of positive RT-PCR test was 45 days (range 3–78 days). Amongst the breakthrough infections of doubly-vaccinated HCW, 90.7% were infected with B.1.617.2, 5.3% by B.1.1.7-like, 1.3% by B.1.538. The most commonly reported symptoms were fever (38 °C or higher (82.3% of all cases)), cough (43.4%), myalgia (20.4%), and loss of smell/taste (14.2%).

Whole genome sequencing was used to test nose and throat swab samples. In hospital A, 66 sequences with high quality whole genome coverage of > 90% were generated, including 43 cases of breakthrough infection. In hospital B, high quality genome sequences were generated from 52 samples, including all 32 symptomatic breakthrough infections.

### Community sequencing

**Figure 1:**
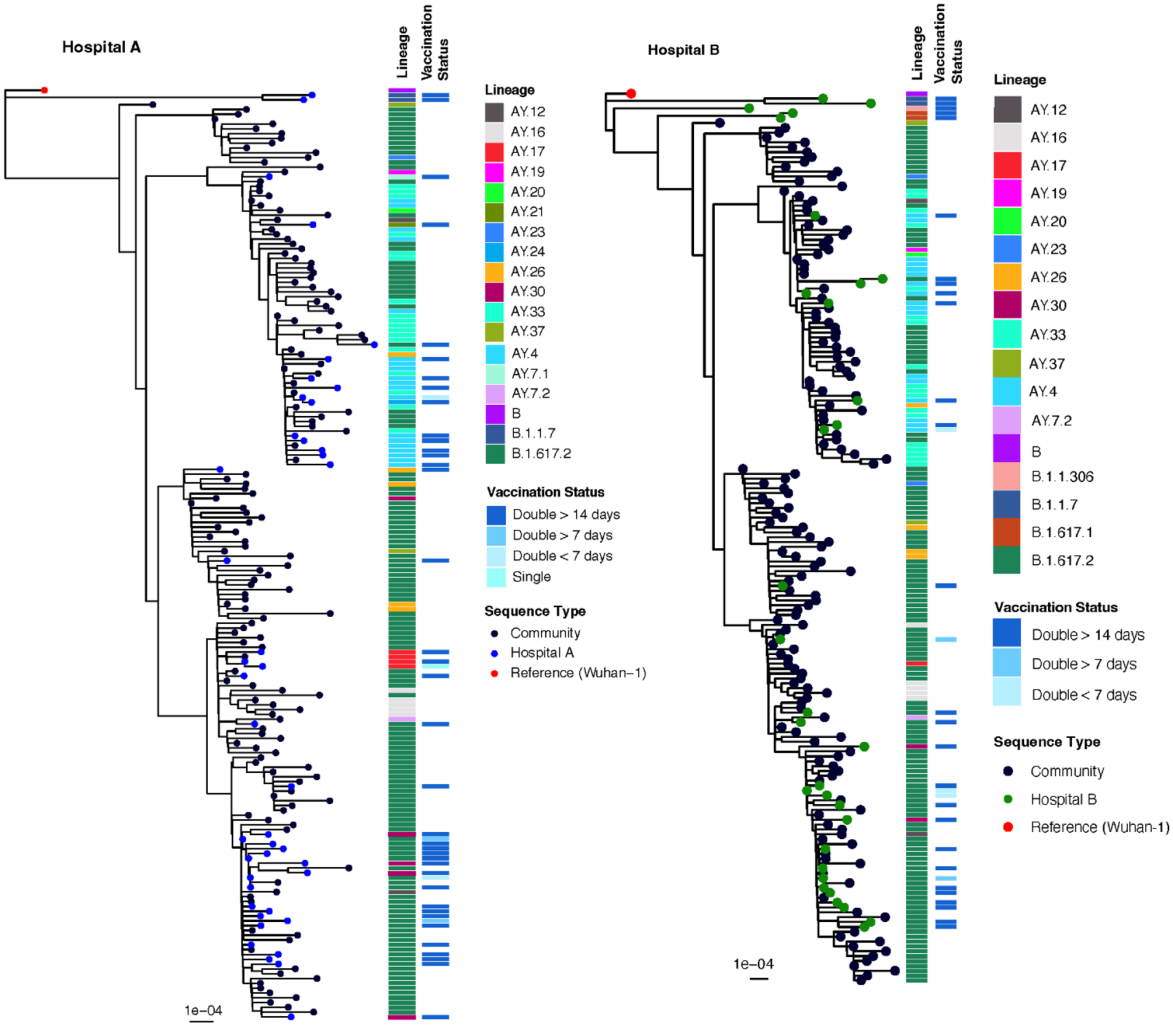
Maximum-likelihood phylogenies of double-vaccinated individuals in Hospitals A and B, with 100 randomly selected community-origin Delta variant SARS-CoV-2 genomes from the state of Delhi, India. Pangolin lineages of all sequences are indicated by the adjacent heatmap. Vaccination status of HCW is indicated by the far-right heatmap. Community sequences have unknown vaccination status.

Between April–May 2021, > 99% of SARS-CoV-2 sequences in India were assigned to the Delta (B.1.616.2) lineage. However, as of August 2021, Delta sub-lineages proliferated—predominantly AY.12—across India and elsewhere^29^. Since then, the Omicron (BA.1 and BA.2) lineages have superseded this. To determine the relationship between community and HCW sequences, we inferred a maximum-likelihood phylogeny to estimate dispersion (Fig. 1) amongst HCWs and the community. The analysis suggested multiple introductions into hospitals A and B, with subsequent intra-hospital transmission. We found significant partitioning of the inferred phylogeny, separating the sequences into distinct clades. Mutations relative to the Delta consensus that were found in these cases were spread across the SARS-CoV-2 genome. None of the identified SNPs were in homoplasic regions of the genome. We note that the individuals 115, 127, and 305 in hospital A had identical virus genomes.

### Characterisation of Delta variant infection dynamics

As a preliminary step towards an analysis of virus transmission we used published data to parameterise distributions describing the incubation period and the infectivity profile of the Delta variant^30^. Data describing the incubation period and the serial interval of the virus were taken from a study of an outbreak of Delta variant SARS-CoV-2 in Guangdong province and are described here. Following previous approaches to characterising infection dynamics^31–33^ we inferred parameters describing the time t_S_ to symptom onset of the virus as a lognormal distribution

$${P}_{LN}\left({t}_{S}|\mu ,\sigma \right)=\frac{1}{x\sigma \sqrt{2\pi }}\mathrm{exp}\left(-\frac{{\left(ln\,{t}_{S}-\mu \right)}^{2}}{2{\sigma }^{2}}\right)$$

with parameters μ = 1.39599 and σ = 0.41354, and the infectivity profile of the virus as an offset gamma distribution

$${P}_{\gamma }\left(t|a,b,o\right)=\frac{{b}^{a}{\left(t-o\right)}^{a-1}\mathrm{exp}\left(-b\left(t-o\right)\right)}{\Gamma \left(a\right)}$$

with parameters α = 38.4805, β = 0.468049, and o = 20.

### Linkage between vaccinated HCW and transmission networks

Integrating our derived parameters into the A2B-COVID^24^ software package, we identified a number of putative transmission pairs between HCWs. The A2B-COVID package combines dates of symptom onset with virus genomes to identify pairs of individuals for whom the data are consistent with direct transmission. Within our data we identified 35 putative transmission events involving 14 HCWs in hospital A, and 26 putative events involving 13 patients in hospital B (Supplementary Figs. 1, 2).

**Figure 2:**
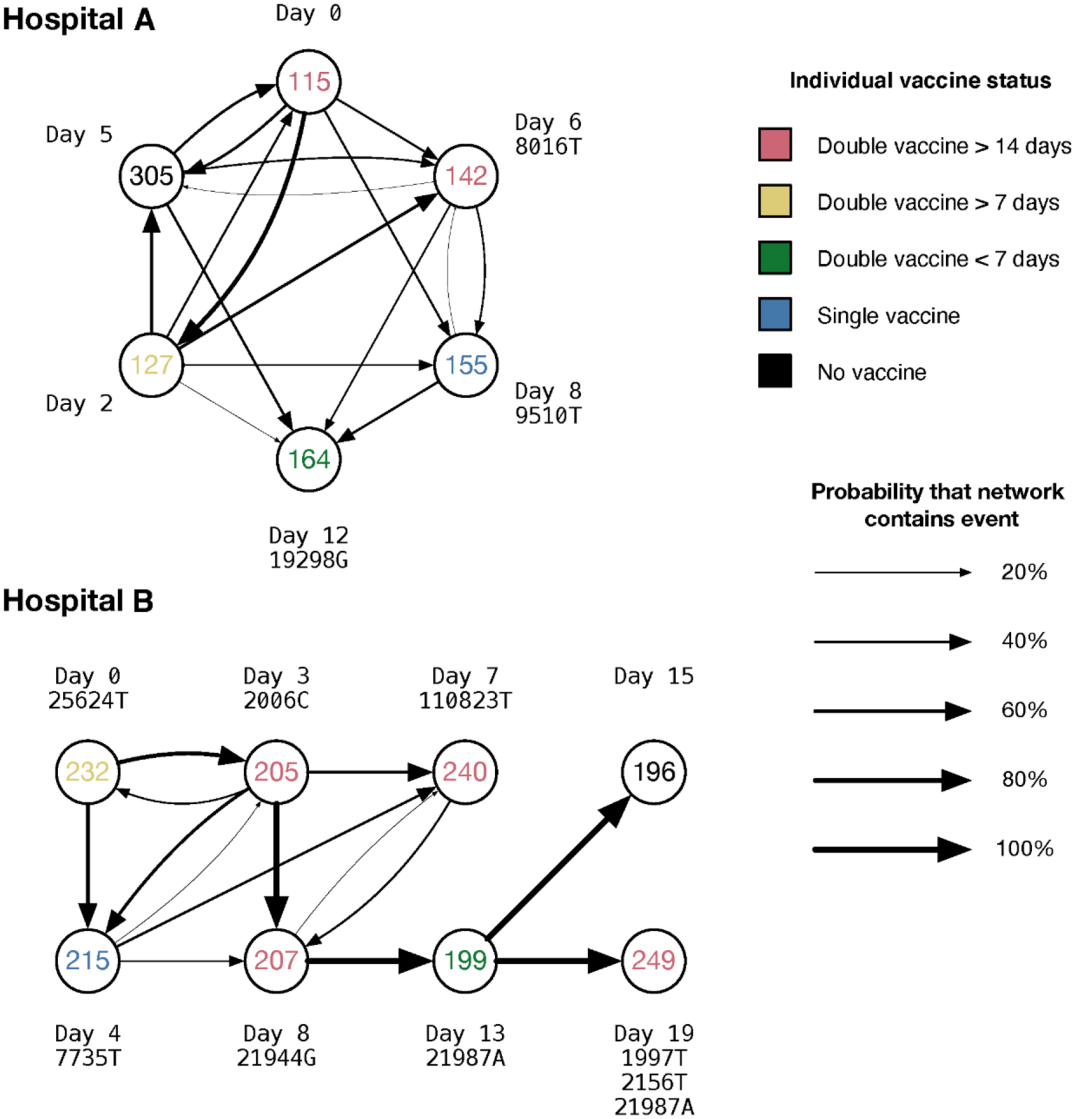
Potential transmission networks between HCWs. Individual labels are coloured according to vaccine status, including the timing prior to infection at which the second vaccine was given, where relevant. The thickness of lines between individuals show the probabilities of distinct pairwise transmission events between individuals; these probabilities are conditional on transmission having occurred between the individuals observed in each network. Labels show the relative dates on which individuals became symptomatic, and respective gains of nucleotides in sequences collected from each individual with respect to the mutual consensus.

Potential transmission networks connecting these cases were inferred using the A2B-Network software package^20^. Given a set of cases infected which are potentially related by transmission, this identifies potential networks of transmission events connecting the cases, and calculates a probability for each specific network, conditional upon the cases being linked by direct transmission. To reduce excessive computational load, potential transmission events in hospital A were filtered to remove those involving the gain of more than one SNP (Supplementary Fig. 3); none of the statistics we go on to derive could have been altered by this step. From the cases remaining we identified a set of possible networks involving six HCWs, two of whom had received their second dose of vaccine at least 14 days prior to reporting symptoms (Fig. 2). A total of 1381 possible transmission networks were identified. Conditional upon these individuals being linked by direct transmission, there was a 92.1% chance that one of the two individuals receiving a second vaccine 14 days prior to infection was the source of transmission to another individual in the network. If this criterion was relaxed to 7 days between second vaccination and infection, we estimated a 99.9% chance that at least one of the three such individuals infected another individual in the network, with a 98.7% probability of direct virus transmission between these three individuals. Other unrelated networks in hospital A were identified but did not involve transmission from vaccinated HCWs to unvaccinated HCWs (Supplementary Fig. 4).

The inferred transmission networks involved a variety of medical staff. In hospital A, a common genome sequence was shared by the junior medical staff member P115, the nursing student P127, and by P305, a member of the nursing staff, who collectively became symptomatic over a period of six days. Three other staff had virus genomes which differed from this by a single SNP each. The ophthalmologic junior medical staff P142 became symptomatic a day after P305; the SNP 8016 T causes a nonsynonymous change in ORF1ab, A2584V. P155 was also a member of the nursing staff; the SNP C9509T leads to the nonsynonymous substitution T3082I in ORF1ab. Finally, P164 was a paramedic; the SNP A19298G represents a nonsynonymous change in the ORF1ab (Y6345C).

An analysis of potential transmission events in hospital B identified a network involving eight HCWs, four of whom had received their second dose of vaccine at least 14 days prior to reporting symptoms (Fig. 2). A total of 128 possible transmission networks between these individuals were identified, all of which implied that one of the four individuals who received a second vaccine at least 14 days prior to infection was the source of transmission to another individual in the network. Among the five individuals who were infected more than seven days post second vaccination, we found a 97.8% chance of a transmission event having occurred between individuals vaccinated with two doses. In hospital B, the individuals P205, P207, and P240 all received their second dose of vaccine at least 14 days before testing positive. P205 was a paediatrician, P207 and P240 both adult physicians. Further details of symptoms, job titles, symptoms, and positive test dates, where known, are shown in Table 2. Full details of SNPs are given in Supplementary Figs. 5 and 6.

**Table 2:** Details of cases in the inferred transmission networks.

Individual	Job description	Symptom onset DD/MM/YY	Test date DD/MM/YY	CT value	Symptoms
**Hospital A**					
P115	Junior medical staff	12/04/21	13/04/21	22.8	Fever, myalgia, sore throat, abdominal cramps
P127	Nursing student	14/04/21	15/04/21	35.2	Throat irritation
P305	Nursing staff	17/04/21	19/04/21	25	Anosmia, conjunctivitis, rhinorrhoea
P142	Junior medical staff (ophthalmology)	18/04/21	19/04/21	29.6	Fever, cold cough
P155	Nursing staff	20/04/21	21/04/21	29	Rashes, fever, myalgia, headache
P164	Paramedic	24/04/21	25/04/21	21.1	Fever, cough, sore throat
**Hospital B**					
P232	Medical officer	09/04/21	12/04/21	15.5	Fever, rhinorrhoea, sore throat
P205	Paediatrician	12/04/21	19/04/21	17.1	Fever, myalgia, anosmia, ageusia
P215	Chief health director/Physician	13/04/21	14/04/21	18.5	Fever
P240	Doctor (pathology labs, COVID wards)	16/04/21	17/04/21	12.8	Fever, cough, myalgia
P207	Physician	17/04/21	19/04/21	16.8	Cough, rhinorrhoea, sore throat
P199	Physician	22/04/21	22/04/21	16.3	Fever, myalgia
P196	OT Assistant	24/04/21	26/04/21	14.6	Fever, sore throat, myalgia
P249	Nursing staff	28/04/21	28/04/21	14.6	Fever, rhinorrhoea

**Figure 3:**
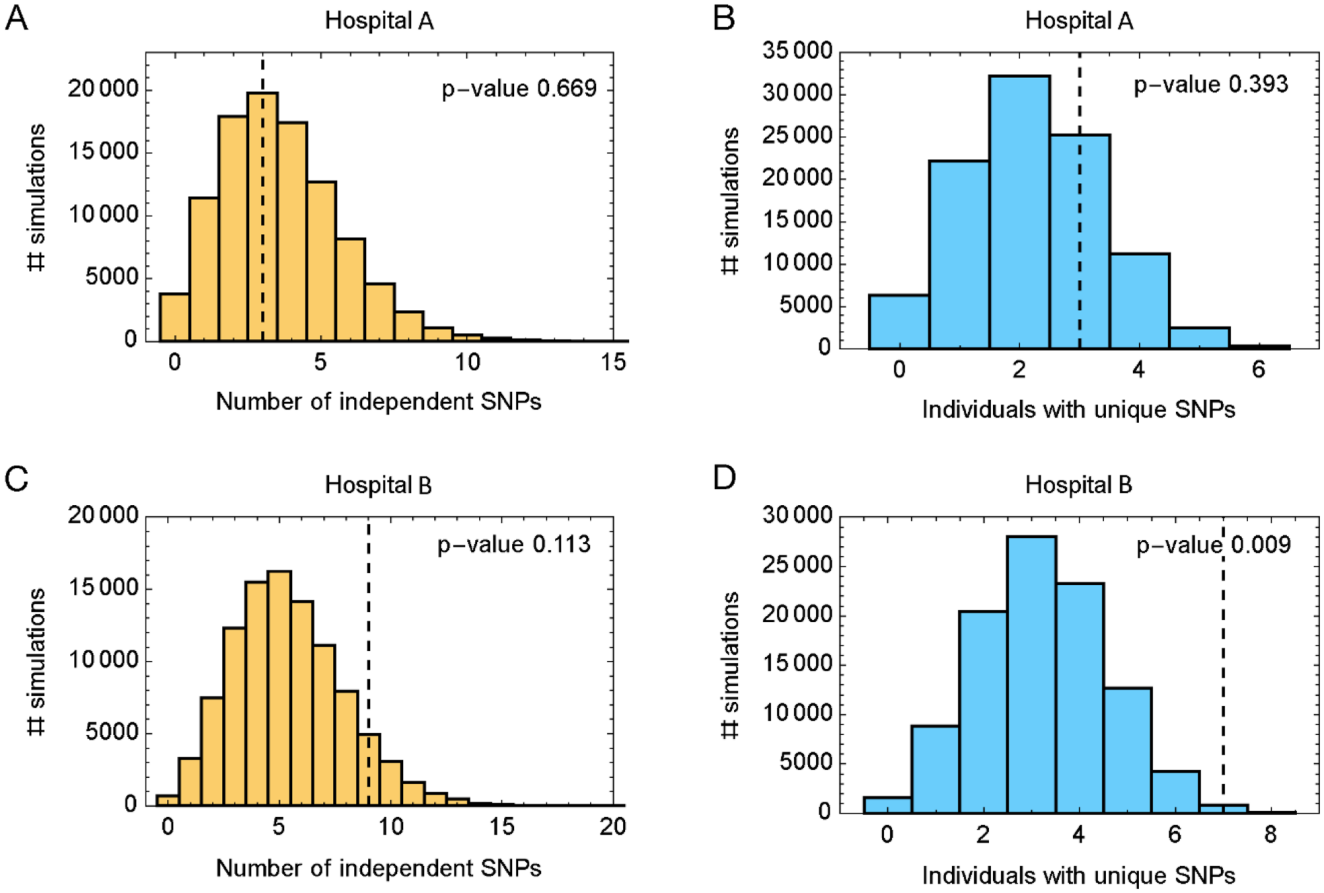
Assessments of network consistency. Data from simulations conducted on the transmission networks inferred for (**A**, **B**) hospital A and (**C**, **D**) hospital B. Simulations used the evolutionary model for the Delta variant of SARS-CoV-2 used to infer transmission networks. Simulated data were assessed to measure the total number of independent SNPs arising across the course of the transmission network, and the number of individuals in the network with SNPs that were unique to themselves. Histograms show distributions of each statistic across simulations, while the vertical dashed line shows the statistic calculated for the inferred network. A high value within the inferred network suggests the likely presence of missing data. Thus, data from the networks inferred for hospital A were consistent with the statistics of the simulations, though the data from hospital B showed  a significant outlier in terms of the number of individuals in the network with unique SNPs. These results provide evidence that the networks derived for hospital B is affected by missing data, though no such conclusion could be made for the networks derived for hospital B.

A simulation-based validation procedure, used to assess the potential influence of missing data upon the derived networks, suggested that the network inferred using data from hospital A was self-consistent, but that the network inferred for hospital B was likely affected by missing data (Fig. 3).

## Discussion

Given the substantial global influence of the Delta variant ^46^ and the more recent Omicron variant ^47,48^, information on transmission between partially- and fully-vaccinated individuals is needed to inform the need for future infection control and vaccination boosting strategies. Our study highlights the potential for Delta variant transmission in a healthcare setting from, and between fully-vaccinated individuals. In the majority of transmission events described here, infections spread from unvaccinated, partially vaccinated and very recently vaccinated HCWs to those who had been fully vaccinated for > 14 days. We suggest that fully-vaccinated HCWs are therefore less likely to infect others. Since writing, several other studies have also highlighted vaccine breakthrough in both community and clinical settings; these literature are broadly divided into those where there has been vaccinated individuals becoming infected^5^ vs. transmission occurring from breakthrough cases^34–36^. Despite vaccine breakthrough, we and others have shown that vaccination is still an effective method of reducing moderate-severe disease^5,37–39^.

The use of in silico predictions of transmission, overlaid with epidemiological investigation data has allowed for targeted infection control and reduction of the risk of transmission between HCWs. Indeed, quickly identifying linked cases of infection is a critical component of the public health response to viral infectious diseases^40^. From a clinical perspective, rapid assessment is required to determine if infection cases have been introduced independently to wards, or if they are potentially linked via direct transmission. Viral genome sequence data provide excellent value in making these assessments and by combining data on infection dynamics, movement data of patients and evolutionary analysis of genome sequences we are able to determine if infection cases are consistent with direct transmission events.

Our study has some caveats: as sequencing was limited to symptomatic cases, we therefore excluded asymptomatic HCWs or those who failed to report their infection status, or patients who could have been the source of some infections. This means that potential transmission events are limited to chains of ≤ 4 events. This highlights the need to sequence both asymptomatic cases and an increased number of patients where an outbreak is occurring. Secondly, the number of viral genomes included in the transmission analysis was limited by incomplete genome coverage. Incomplete sequences do not provide enough data for accurate evolutionary analysis and therefore were excluded from transmission reconstruction. This likely resulted in incomplete transmission chains where the intermediary was not identified, hence the strict SNP threshold of one was used. The algorithm used in our study for network reconstruction builds networks only from the cases of infection that are known to have occurred. In our dataset, while symptomatic HCWs were surveyed for infection, there is the potential that asymptomatic HCWs, or patients could form unobserved parts of the true transmission networks. Due to limitations in resources, we were unable to conduct a full epidemiological investigation alongside symptomatic screening—this would strengthen any findings and allow us to capture a more complete picture of transmission. However, we compared the networks derived from hospital data to ensembles of simulations describing identical networks of transmission events, comparing the genetic properties of the hospital networks to the statistics of simulated viral populations; an excess of genetic substitutions in a network, relative to simulations, can indicate the presence of missing parts of its inferred evolutionary history as would arise from missing data (Supplementary Fig. 7).

Calculations suggested that the networks inferred for hospital B are likely to be affected by missing data, though the data from hospital A was not clearly affected. Although the possibility of missing data can never be full discounted, these calculations provide a basic level of validation for the network inferred in the case of hospital A. In other outbreak scenarios, additional sequencing would provide increased resolution and allow for reconstruction of longer transmission chains. Our data were carried out in HCW within hospitals; however, the model is also applicable to community settings, relying on genomics data, and epidemiological data where available. Our study is important in that it describes a novel approach to identifying transmissions that is more rapid and scalable, critically not requiring an infection control infrastructure.

Although vaccination is still highly effective for Delta in protecting against severe disease^39^, we have demonstrated that breakthrough infections still occur in healthcare settings in individuals within 60 days of the second dose when circulating neutralising antibody levels are at their highest^41^. Given the risk of onward transmission to potentially very vulnerable patients (including people in whom vaccination is less effective, such as those with compromised immune systems), our findings highlight the need for ongoing infection control measures even in highly vaccinated populations, in order to limit onward transmission.

## Methods

### Study design

Vaccination of HCWs began in January/February 2021 with the ChAdOx1 vaccine (Covishield). All frontline HCW were encouraged to receive a vaccination and were invited to do so. Shortly after vaccination, additional sequencing capacity was introduced to collect enhanced data. During the wave of infections in March and April 2021, symptomatic SARS-CoV-2 was confirmed amongst 1100 and 4000 HCW staff members, respectively in Hospital A and B. During this period, all frontline, symptomatic healthcare workers from each hospital, were diagnostically tested for the presence of SARS-CoV-2 by means of reverse transcriptase polymerase chain reaction (RT-PCR) using TRUENAT or CBNAAT (GENEXPERT). Findings were expressed as the cycle threshold (Ct) for the gene encoding the nucleocapsid protein (N gene) for hospital A, and the gene encoding for the envelope protein (E gene) for hospital V. A Ct value of less than 30 was considered to be infective. A vaccine breakthrough infection was defined as detection of SARS-CoV-2 in a sample collected from an individual 14 days after receipt of a second dose of ChAdOx1.

Ethical approval for this study and specifically the investigation of vaccine-elicited antibodies in sera from vaccines was obtained from the East of England Cambridge Central Research Ethics Committee, University of Cambridge, Cambridge, United Kingdom (REC ref. 17/EE/0025). Studies involving HCWs (including testing and sequencing of respiratory samples) were reviewed and approved by The Institutional Human Ethics Committees of the National Centre for Disease Control (NCDC) and Council Of Scientific And Industrial Research–Institute Of Genomics And Integrative Biology, India (CSIR IGIB) (NCDC/2020/NERC/14 and CSIRIGIB/IHEC/202021/01). Participants provided informed consent.

All methods followed in this manuscript were performed in accordance with the relevant guidelines stipulated by the ethical approval committee, as reviewed.

### Bioinformatics and phylogenetic analysis

Fasta consensus sequences were obtained from two separate Hospitals in Delhi, India. All sequences were concatenated into a single fasta and aligned to reference strain MN908947.3 with mafft v7.847^42^ using the –keeplength and –addfragments options. Following this, all sequences were screened for number of gaps and N-regions using the Nextclade v1.5.4 (https://clades.nextstrain.org/) server. All sequences were assigned a lineage with Pangolin v3.1.11^43^, pangoLEARN (dated 9th August 2021) and scorpio v0.0.14. Sequences that could not be assigned a lineage were discarded. After assigning lineages, all sequences with more than 5% N-regions were also discarded.

To contextualise outbreak sequences, all sequences from India with lineage defined as B.1.617.2 from the month of April 2021 were downloaded from the Global Initiative on Sharing Avian Influenza Data (GISAID) EpiCoV database. Incomplete (< 29,000 base pair), duplicate, and low-quality sequences (defined as equal to or more than 5% Ns; less than 95% genome coverage) were excluded from further analysis.

### SNP distance, variant calling and annotation

Single-nucleotide polymorphisms (SNPs), relative to the Delta variant strain, were identified by re-alignment of groupings of sequences based on each hospital to the Delta variant consensus reference (MZ359841.1) using mafft v7.8477 with the –keeplength and –addfragments parameters. Initial analysis of pairwise SNP distance between each patient was conducted using snp-dists v0.8.2 with default parameters.

Following this, a VCF of acquired mutations of each patient with respect to the reference strain MN908947.3 (Wuhan-Hu-1) is calculated by snp-sites v2.5.1^44^ using the -v and -c option. Multiallelic variants are broken down into biallelic variant representations and are subsequently annotated by snpEff v5.0e^45^ with reference to MN908947.3. The nucleotide and amino acid variants between each transmission pairs are extracted from the VCF, and SNP number verified using an in-house script (https://github.com/TKMarkCheng/Indian_HCW). The VCFs were manually scanned to identify homoplasic/problematic sites (https://github.com/W-L/ProblematicSites_SARS-CoV2).

### Estimating transmission dynamics for the Delta variant

In order to evaluate potential transmission events involving the Delta strain of SARS-CoV-2 we used data published on an earlier transmission dynamics study involving Delta, to re-derive parameters for these two distributions^30^. In that study, based on 68 infections with clear chains of transmissions, generation times and serial intervals were calculated by Zhang et al., and were used in our model to reflect Delta dynamics more accurately.

#### Incubation period of the virus

The incubation period of the virus is modelled in A2B-COVID by a lognormal distribution, described as follows:

$${P}_{LN}\left(x|\mu ,\sigma \right)=\frac{1}{x\sigma \sqrt{2\pi }}\mathrm{exp}\left(-\frac{{\left(ln\,x-\mu \right)}^{2}}{2{\sigma }^{2}}\right)$$

Zhang et al. report a mean for the incubation period of 4.4 days, with a standard deviation of 1.9 days^30^. We note that the mean and standard deviation of x in the lognormal distribution are given respectively by

$$\bar{x}=\mathrm{exp}\left(\mu +\frac{{\sigma }^{2}}{2}\right)$$

and

$$Std\left(x\right)=\left(\mathrm{exp}\left({\sigma }^{2}\right)-1\right)\mathrm{exp}\left(2\mu +{\sigma }^{2}\right)$$

These equations allow for numerical solution, which was performed using the Mathematica software (Wolfram Research Illinois, USA, https://www.wolfram.com/mathematica/) package v. 13.0.1, providing the values μ = 1.39599 and σ = 0.41354.

#### Serial interval

The serial interval (time between symptom onsets) is modelled by He et al. using an offset gamma distribution^31^, with parameters a, b, and o.

$${P}_{\gamma }\left(x|a,b,o\right)=\frac{{b}^{a}{\left(x-o\right)}^{a-1}\mathrm{exp}\left(-b\left(x-o\right)\right)}{\Gamma \left(a\right)}$$

Zhang et al. report a mean for this distribution of 2.3 days, with a standard deviation of 3.4 days^30^. We note that the mean and standard deviation of this distribution are given by

$$\bar{x}=ab+o$$

and

$$Std\left(x\right)=a\sqrt{b}$$

These equations allow for straightforward solution. Here the offset is included to account for negative intervals, whereby individual A infects individual B, but B becomes symptomatic before A. Setting the value of the offset o to 20 days (changing this does not greatly affect the shape of the final distribution), the mean and standard deviation are satisfied by the values a = 43.0182 and b = 0.518386.

#### Infectivity profile

Finally, to derive an infectivity profile for the Delta strain of SARS-CoV-2, assuming it to follow an offset gamma distribution with parameters α, β, and the offset o = 20 days. We used numerical methods to find parameters α and β so as to minimise the distance metric

$$D=\sqrt{{\sum }_{x=-40}^{30}{\left[{P}_{\gamma }\left(x|a,b,o\right)-{\int }_{-\infty }^{\infty }{P}_{LN}\left(x-y|\mu ,\sigma \right){P}_{\gamma }\left(y|\alpha ,\beta ,o\right)dy\right]}^{2}}$$

In other words, we derived parameters for an infectivity profile such that the compound of it and the distribution describing the incubation period of the virus provided the closest possible fit to the inferred serial interval. From this calculation we obtained the values α = 38.4805 and β = 0.468049. Inferred distributions are shown in Supplementary Fig. 8.

### Identifying plausible cases of person-to-person transmission

As an initial assessment of whether participants in the study had passed an infection to another, we utilised A2B-COVID^24^. This software considers data from individuals in a pairwise fashion, considering the timing of symptom onset and virus genome sequences in order to assess for each pair of individuals A and B whether the data are consistent with an underlying model of direct virus transmission from A to B. Data from each pair are described as ‘consistent’ with transmission, ‘borderline’, or ‘unlikely’ to have been produced given direct transmission.

### Estimating likelihood of person-to-person transmission

Network reconstruction was performed using the A2B-Network software^20^. Again using dates of symptom onset and virus genome sequences, this identifies plausible networks via which all of the individuals in a set could have transmitted the virus between themselves, and assesses the probability of each such network having occurred, given our model assumptions. In this manner, the code produces ensembles of networks, describing the extent to which the data constrain the possible routes of transmission of the virus. The probabilities we report were calculated by summing network likelihoods. For example, the probability that a network contains a transmission event between doubly vaccinated individuals is the sum of the probabilities of the networks which contain at least one such transmission event. Our model assumes a set of underlying parameters describing the transmission dynamics and rate of evolution of the SARS-CoV-2 virus. The network probabilities we report are conditional on these parameters and upon the assumption that the individuals to whom we apply the model are connected by a transmission network.

### Network validation

Transmission networks were validated for self-consistency using a model of simulated SARS-CoV-2 outbreaks. Simulations used identical parameters to the network inference model to describe the transmission dynamics and rate of evolution of the SARS-CoV-2 virus, and exploited the fact that missing data can lead to missing time in the evolutionary tree (Supplementary Fig. 7). A total of 10^5^ networks were chosen at random from the ensemble of networks inferred for the hospitals, with the probability that a specific network was chosen being identical to its probability within the inference. Across these networks we then calculated distributions of the total number of unique mutations, which evolves proportional to the time within the transmission tree, and the number of individuals with mutations observed in no other individual, which measures the time in the tree after the last transmission from an individual to someone else.

## Supplementary Information

Supplementary Figures.

## Data Availability

All fasta sequences and in-house scripts used to extract and verify VCF and SNPs are available from https://github.com/TKMarkCheng/Indian_HCW. Data is also freely available for download from https://gisaid.org.
