# Supplementary material for: Transmission of B.1.617.2 Delta variant between vaccinated healthcare workers
Source: Sci Rep. 2022 Jun 21;12:10492. doi: 10.1038/s41598-022-14411-7 (PMC9212198; doi:10.1038/s41598-022-14411-7)
Supplement: Supplementary file 1 — Supplementary Figures. [file 41598_2022_14411_MOESM1_ESM.pdf]

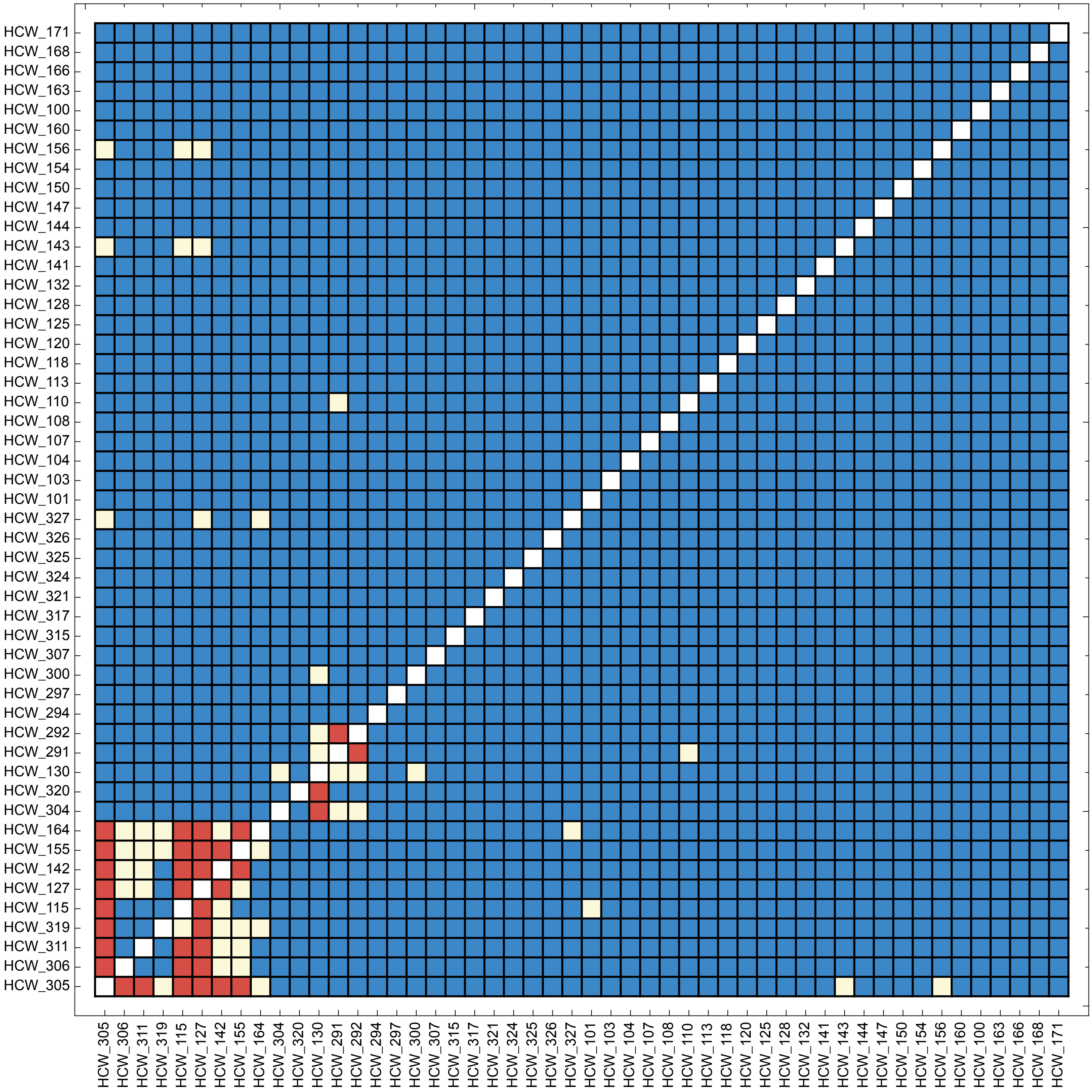

Supplementary Figure 1. Output from A2B-COVID applied to data from hospital A. A red square shows that the data was consistent with a pairwise transmission event having occurred between the two corresponding individuals (from the individual labelled on the vertical axis to the one on the horizontal axis). Yellow squares indicate borderline cases, while blue squares show cases in which direct transmission was unlikely.

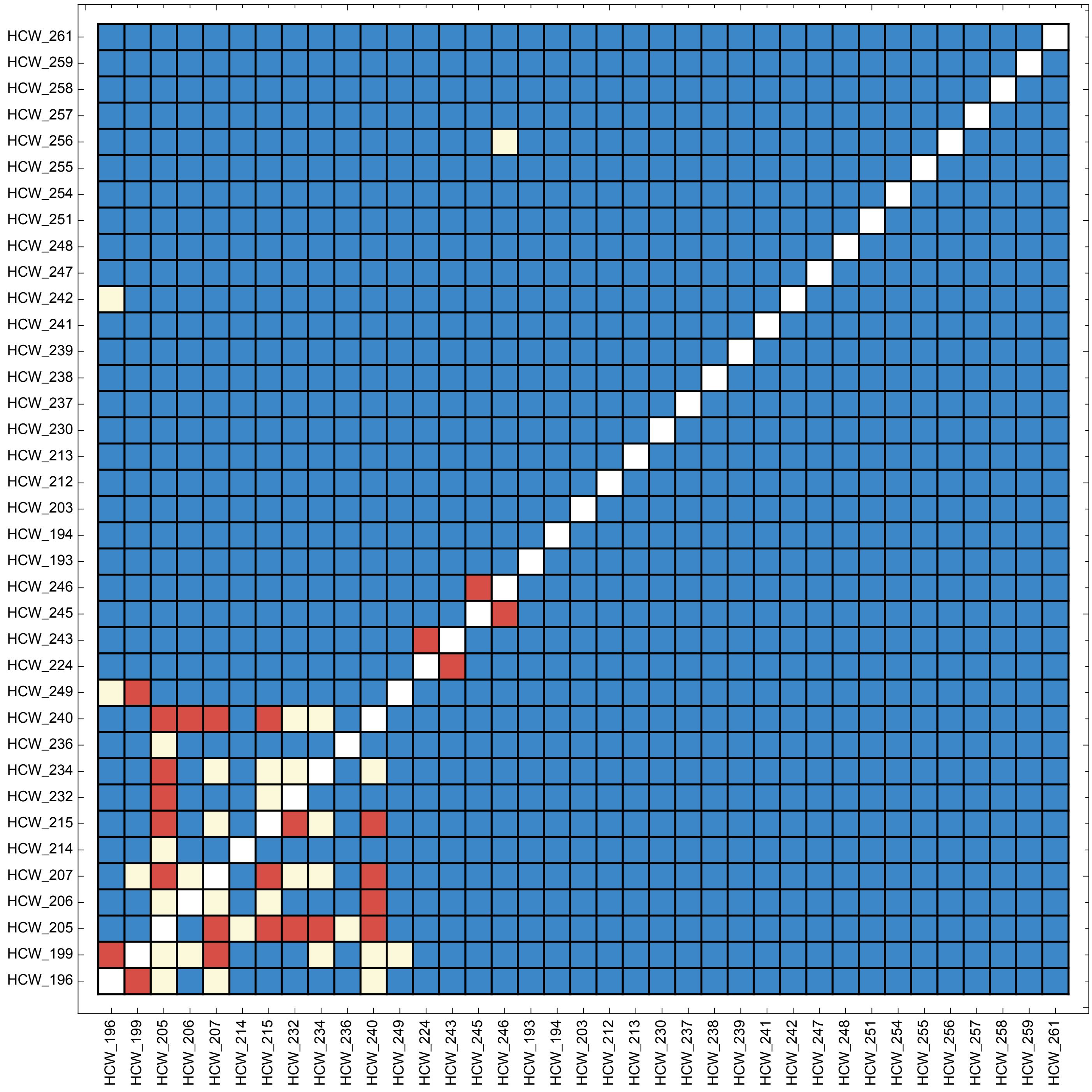

Supplementary Figure 2. Output from A2B-COVID applied to data from hospital B. A red square shows that the data was consistent with a pairwise transmission event having occurred between the two corresponding individuals (from the individual labelled on the vertical axis to the one on the horizontal axis). Yellow squares indicate borderline cases, while blue squares show cases in which direct transmission was unlikely.

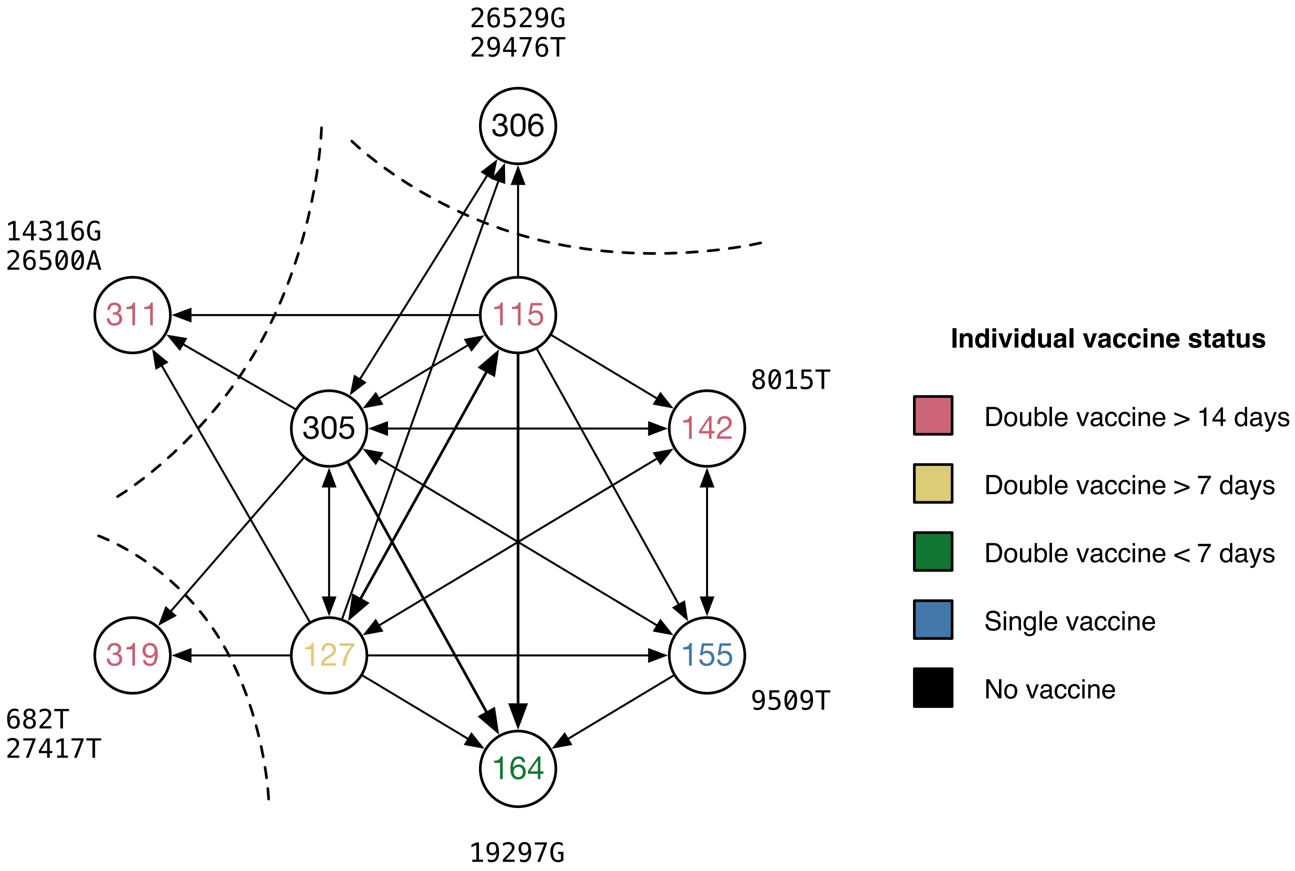

Supplementary Figure 3. Network of possible transmission events between individuals in hospital A, inferred using the A2B-Network software. Individual labels are coloured according to vaccine status, including the timing prior to infection at which the second vaccine was given, where relevant. Relative gains of nucleotides are shown with respect to the mutual consensus. Dashed lines show individuals excluded from further analysis. None of the excluded individuals could have transmitted to any of the included individuals, such that the pruning of the network does not affect our calculations of who may have infected whom.

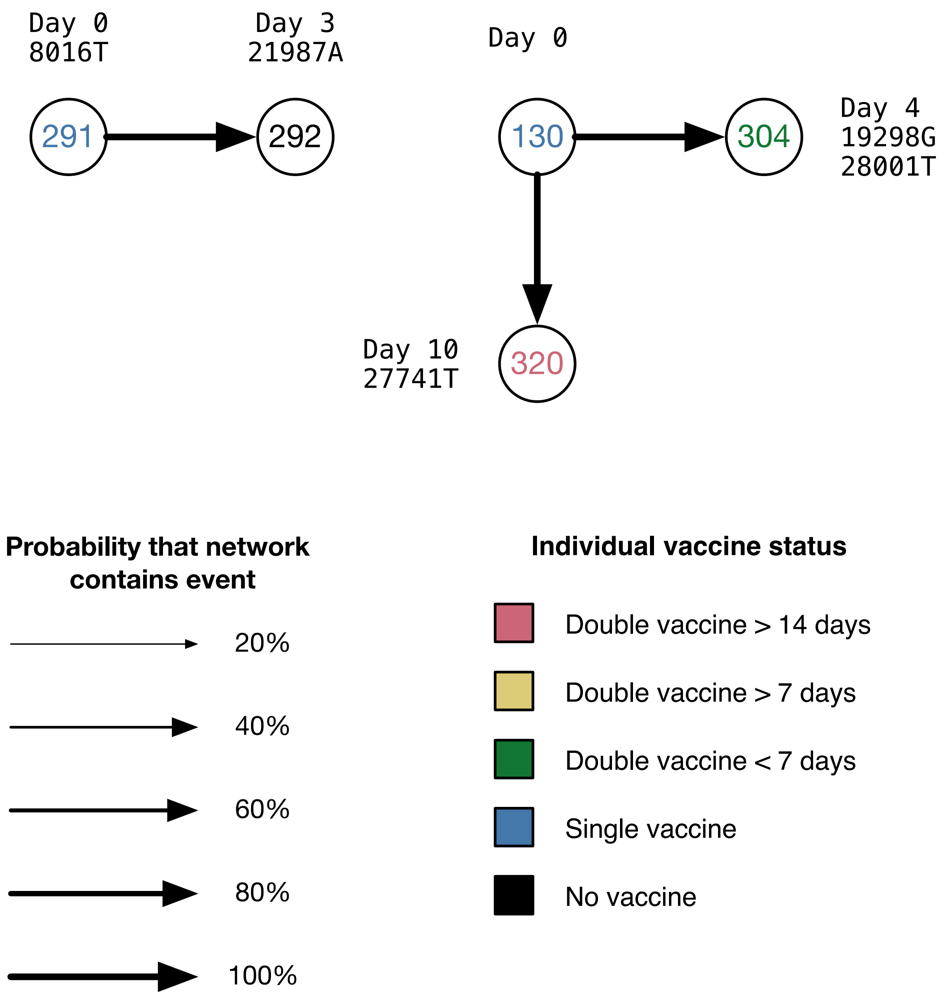

Supplementary Figure 4. Other potential transmission networks identified in hospital A by the A2B-Network software. Individuals labelled in red text had been vaccinated at least 14 days before becoming symptomatic for infection, while those in black text had either received fewer doses of vaccine or had received their second dose more recently than 14 days. The thickness of the lines shows the probability of the reconstructed network containing the event shown by an arrow. Network probabilities are conditional on transmission having occurred between these observed individuals. Labels show relative gains of nucleotides with respect to the mutual consensus. Dashed lines show individuals excluded from further analysis.

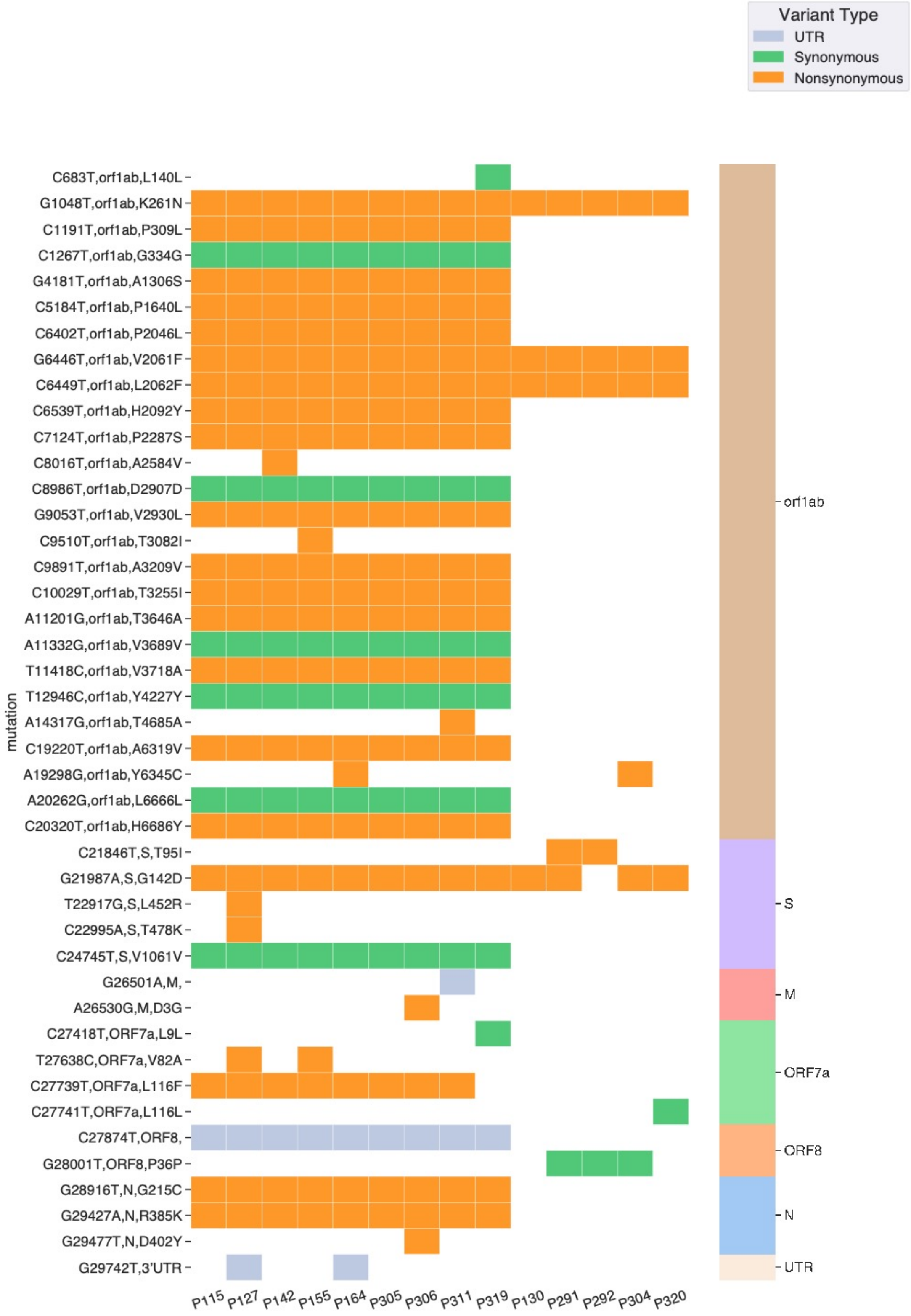

Supplementary Figure 5. SNPs identified in participant viral consensus sequences in hospital A. SNPs identified are with respect to Delta strain (MZ359841.1). Gene location shown on right. Heatmap indicates synonymous, non-synonymous variants, and variants located within untranslated regions.

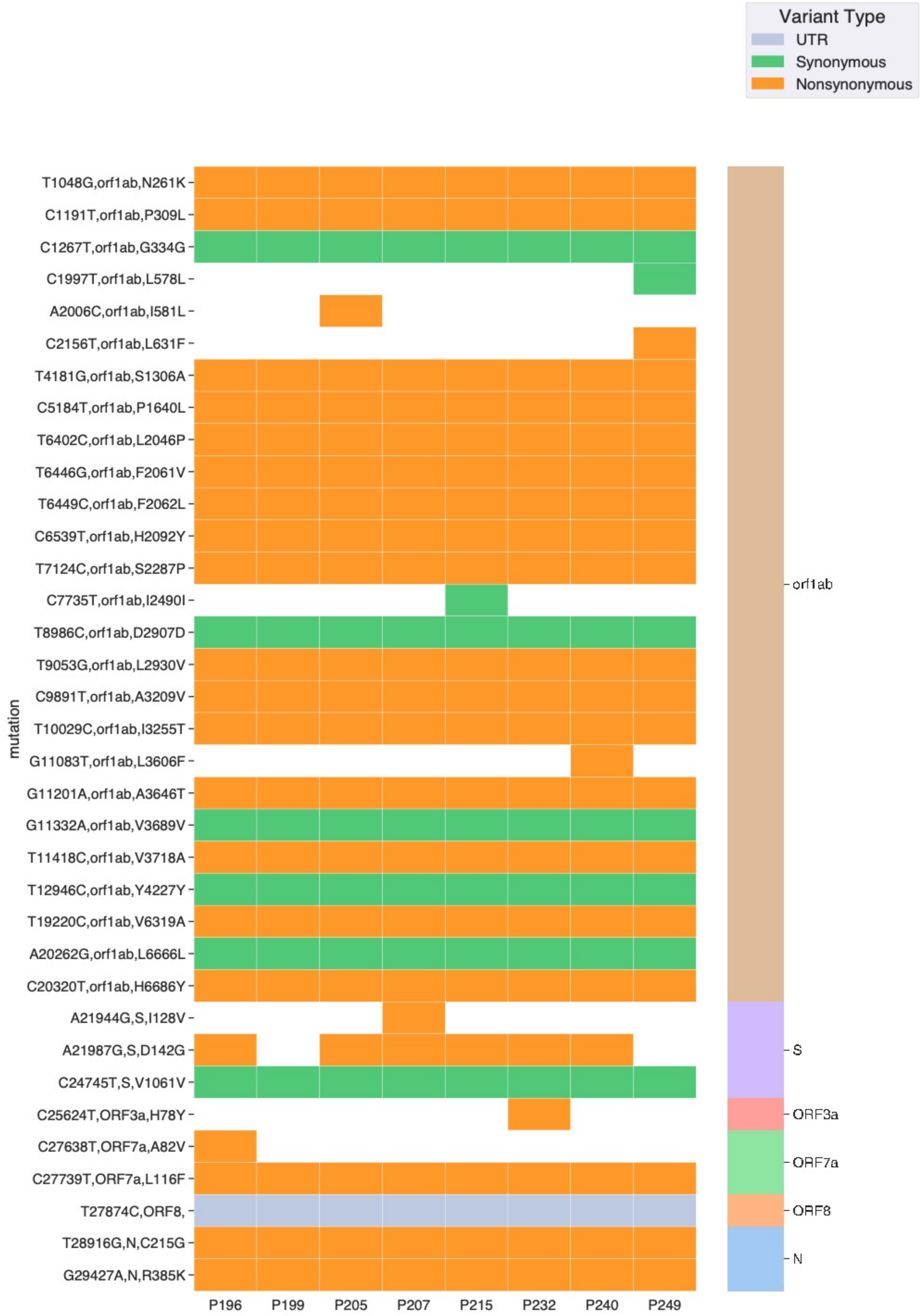

Supplementary Figure 6. SNPs identified in participant viral consensus sequences in hospital B. SNPs identified are with respect to Delta strain (MZ359841.1). Gene location shown on right. Heatmap indicates synonymous, non-synonymous variants, and variants located within untranslated regions.

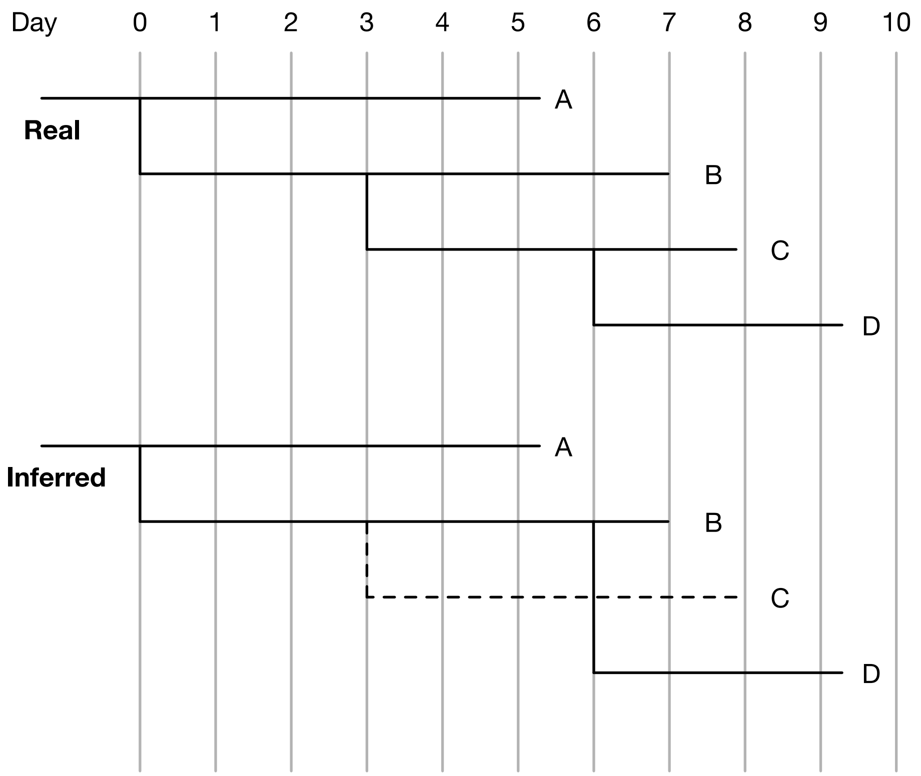

Supplementary Figure 7. Missing time in evolutionary trees arising from missing data. In the case above, transmission events happen on days 0, 3, and 6 between individuals A, B, C, and D. Within the real transmission tree, on day 7 of the outbreak the viruses in individuals B and D are separated by eight days of evolution, four in individual B, with another four first in C then in D. In the inferred transmission tree, data from individual C is missing. If transmission is inferred to have occurred from B to D on days 6, then only two days separate the viruses in individuals B and D. Thus the inferred network contains less time for the virus to evolve than the real network. This allows for a test of network consistency; if sequences from the observed individuals A, B, and D are separated by more substitutions than can be explained by the proposed network, this may indicate the presence of missing data.

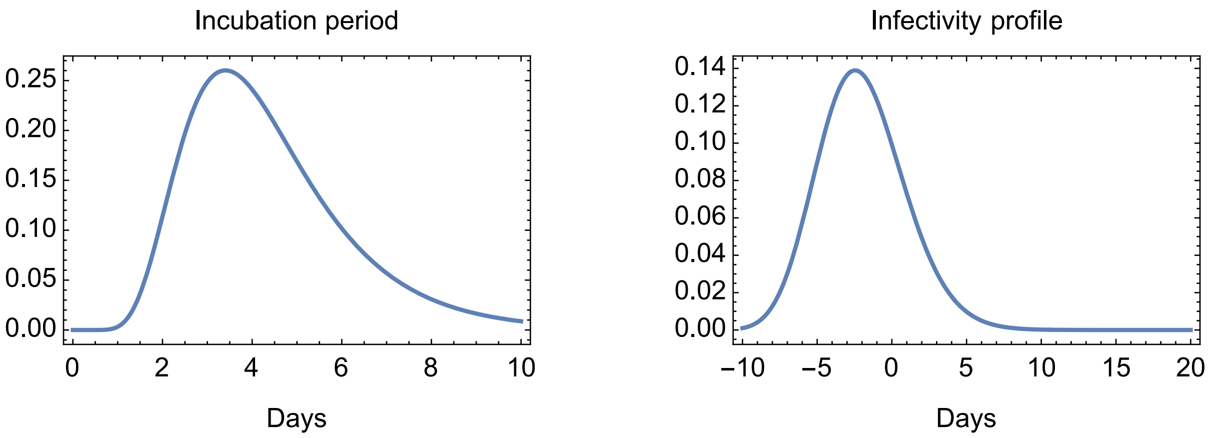

Supplementary Figure 8. Inferred distributions for the incubation period and the infectivity profile of the B.1.167.2 or Delta variant of SARS-CoV-2, used in our analysis.
